# Intermittent hypoxia index: a new indicator for assessing the degree of intermittent hypoxia in obstructive sleep apnea

**DOI:** 10.3389/fmed.2025.1400376

**Published:** 2025-04-29

**Authors:** Kui Xie, Xiaoqing Tang, Jiacheng Zhou, Xiang Liu, Yunyun Zhang, Xiaochuan Cui

**Affiliations:** ^1^Department of General Practice and Sleep Center, The Affiliated Wuxi People's Hospital of Nanjing Medical University, Wuxi People’s Hospital, Wuxi Medical Center, Nanjing Medical University, Wuxi, China; ^2^Advanced Information Materials Research Center, School of Physics and Materials Science, Guangzhou University, Guangzhou, China

**Keywords:** intermittent hypoxia, Newton quadrature low oxygen load assessment system, intermittent hypoxia index, obstructive sleep apnea, Newton-cotes formula

## Abstract

**Objective:**

In order to objectively and accurately evaluate the degree of nocturnal intermittent hypoxia (IH) in patients with obstructive sleep apnea (OSA), we developed the Newton quadrature low oxygen load assessment system (NLAS) to seek a new, quantifiable, comprehensive evaluation index of intermittent hypoxia (intermittent hypoxia index, IHI).

**Methods:**

Demographic characteristics, anthropometric measurements, and polysomnography (PSG) parameters [oxygen desaturation index (ODI), lowest oxygen saturation (LSpO_2_), time below 90% saturation (T90)] of 732 patients with OSA who underwent multi-channel sleep monitoring at the Sleep Center of Affiliated Wuxi People’s Hospital, Nanjing Medical University, from 2019 to 2023 were retrospectively collected. The IHI was calculated using the NLAS (Certificate of Registration Number for Computer Software Copyright of the People’s Republic of China: 12208933), and its threshold was defined. Additionally, correlation analysis was performed between IHI and T90, LSpO_2_, and ODI.

**Results:**

Among the 732 patients with OSA, IHI showed significant correlations with T90 (*r* = 0.922) and LSpO_2_ (*r* = 0.866), and moderate correlation with ODI (*r* = 0.675). The threshold for diagnosing hypoxia in OSA patients using IHI was 7.178 (%s/min).

**Conclusion:**

This study demonstrates that IHI calculated using NLAS covers various dimensions of IH indices in OSA patients undergoing multi-channel sleep monitoring. It correlates with parameters such as T90, LSpO_2_, and ODI. Independent of existing IH assessment indices, IHI holds promise as a new comprehensive assessment index for evaluating the degree of nocturnal IH in OSA.

## 1 Introduction

According to epidemiological statistics, one billion people worldwide suffer from obstructive sleep apnea (OSA) (1). OSA is associated with many comorbidities such as hypertension, diabetes, arrhythmia, pulmonary arterial hypertension, and congestive heart failure (2).OSA is mainly characterized by recurrent episodes of upper airway collapse and obstruction during sleep (3), which can result in repeated hypoxia-reoxygenation of the body, i.e., intermittent hypoxia (IH), which is one of the main mechanisms underlying the pathophysiology of OSA-associated cardiovascular diseases and metabolic syndrome (4, 5). At present, the main indicators used to evaluate IH in OSA are lowest oxygen saturation (LSpO_2_), sleep time with oxygen saturation below 90% (T90), and oxygen desaturation index (ODI). However, these indicators can only reflect a single pathophysiological feature of IH, evaluating only the duration, length or frequency of oxygen reduction, and cannot capture its overall characteristics, thus failing to serve as a comprehensive hypoxia evaluation indicator in clinical practice (6). In order to more accurately assess the severity of IH and its pathophysiological characteristics in OSA patients, Azarbarzin (7) et al. proposed hypoxic burden (HB). However, there is no relevant literature report on HB showing that it can be specifically measured directly from polysomnographic monitoring data. Based on this, we developed the NLAS to calculate the IHI using the Newton-Cotes formula and to assess its correlation with traditional hypoxic assessment metrics.

## 2 Methods

### 2.1 Study subjects

This was a retrospective study. Subjects were selected according to the inclusion criteria: (1) patients who met the diagnostic criteria and efficacy standards for OSA-hypopnea proposed by the American Academy of Sleep Medicine (AASM), and whose apnea-hypopnea index (AHI) was ≥ 5;. (2) Age 18–80 years old, male or female. Exclusion criteria: (1) AHI < 5; (2) unable to obtain original blood oxygen saturation information or complete PSG monitoring information; (3) diagnosed with chronic obstructive pulmonary disease, bronchial asthma, chronic bronchitis, restrictive lung disease, acute respiratory distress syndrome or other respiratory diseases, or having a minimum blood oxygen saturation when awake of less than 90%. Polysomnography data of 732 patients who underwent polysomnography monitoring using the Philips Alice polysomnography device at the Sleep Center of Wuxi People’s Hospital Affiliated to Nanjing Medical University between January 1, 2019 and December 31, 2023 were included in the study. This study was approved by the Clinical Research Ethics Committee of Wuxi People’s Hospital Affiliated to Nanjing Medical University (Ethics Number: KY24016).

### 2.2 Newton quadrature low oxygen load assessment system (NLAS)

NLAS is a computing software program that can be opened to all users after registration and login. For details, see File 1.The NLAS mainly consists of three modules: data collection, data analysis and data storage. The data collection module calculates and displays the duration, depth, and frequency of desaturation and IHI during the monitoring period; the main function of the data analysis module is comparative analysis, which displays the differences between oxygen desaturation index and IHI and the comparison of T90, T85, and T80 duration and oxygen desaturation index and hypoxic area. The data storage module has a memory function that can record all historical uploaded data and store it by time period. Users can click the query button to query. There is a scroll bar at the bottom of any module interface, and the left side shows the time of desaturation episodes, called abnormal results. Dragging the scroll bar or clicking the abnormal results on the left will produce data on the corresponding duration, depth, graphics and other information of desaturation.

**Figure 1:**
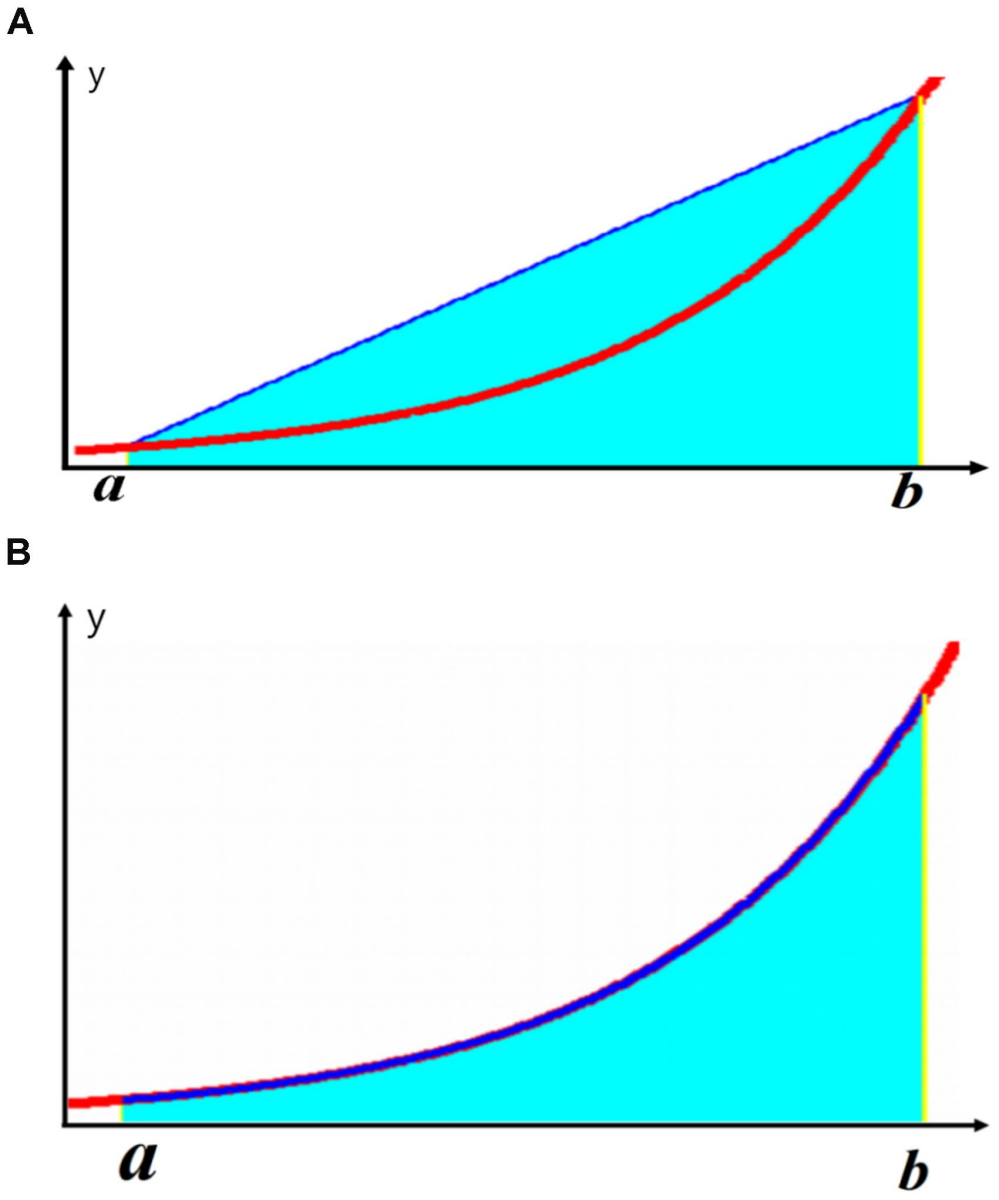
Illustration of different algebraic accuracies: **(A)** Trapezoidal formula; **(B)** Newton-Cotes formula.

Using NLAS, we analyzed the corresponding parameters of nocturnal IH and calculated the IHI value (Table 1). The specific method is as follows: NLAS uses the Newton-Cotes formula to calculate the area under the desaturation curve (If the decrease in oxygen saturation (SpO_2_) was ≥ 3% of the baseline value, it was counted as one desaturation). This area is divided by the total sleep time (TST) in minutes to obtain the IHI (IHI is calculated in Supplementary file 1). In addition to IHI, the NLAS system can also calculate the maximum desaturated area, the average desaturated area, the longest duration, and the average desaturated depth of the patient’s overnight desaturation event, and allows the user to view the time and frequency of the desaturation distribution, the duration of each desaturation event, the degree of oxygen drop and other information. It can provide data support for studying the relationship between the duration of respiratory related events, attack frequency, oxygen drop depth and comorbidities in OSA patients. The Newton-Cotes formula is a commonly-used formula that uses interpolation to calculate numerical integration. It is a special calculation method when the nodes are equidistant with the integration interval equally divided. It reflects the correlation between data and the integration of fluctuations. Because the values are simple by themselves without high-order derivatives, the result error is small. From the following formula, it can be seen that the trapezoidal formula only has first-order algebraic accuracy, while the Newton-Cotes formula can have fifth-order algebraic accuracy (different algebraic precision graphs are shown in Figure 1). Therefore, it has better stability and smaller errors. Unlike Excel and other calculation tools, Excel is only a calculation tool, there is no coding function, can not automatically identify desaturation, if it is used to calculate the hypoxic load, it is necessary to manually identify the graphs and data of each desaturation, and enter the calculation law one by one, and finally excel can calculate the AUC. Then the same piece of data, counted by different technicians will get different then the same data, counted by different technicians will get different results, which will affect the accuracy of the data. It is with this in mind that we have endeavored to solve this problem effectively by developing a calculation software.

**Table 1:** Name and explanation of parameters involved in the Newton assessment system software for hypoxic load.

Parameter name (Unit)	Explanation
Total area (%*s)	The sum of the areas under all desaturation curves, that is, using the Newton-Cotes formula to calculate the area of a single desaturation curve, and then add them up to get the total area
Maximum area (%*s)	The maximum area under a single oxygen descent event
Average area (%*s)	The average area of all oxygen descent events, Average area = Total area / Number of desaturations
Maximum duration(s)	The longest time SPO_2_ < 90% lasts in a single oxygen descent event, i.e., the longest oxygen reduction time
Minimum duration(s)	The shortest time SPO_2_ < 90% lasts in a single oxygen descent event
Average duration(s)	The average time of all oxygen descent events, Average Duration = Sum of all oxygen descent times / Number of Desaturations
Maximum depth (%)	The amplitude at which the SPO_2_ drops from the baseline to the lowest value in a single oxygen descent event, i.e., the maximum oxygen reduction
Average depth (%)	The average amplitude of SPO_2_ drops in all oxygen descent events, Average Depth = Sum of SPO_2_ drop amplitudes in all oxygen descents / Number of desaturations, i.e., average SPO2 drop amplitude
IHI (%s/min)	The total area under the desaturation curve calculated by NLAS using the Newton-Cotes formula divided by the total sleep time in minutes. The total area under the desaturation curve is calculated by the Newton-Cotes formula

Interpolating integration formula (8):

$$\int_{a}^{b}f\left(x\right)dx\approx \int_{a}^{b}P\left(x\right)dx=\overset{n}{\underset{k=o}{\sum }}A_{k}f\left(x\right)$$

$$\int_{a}^{b}f\left(x\right)dx\approx \left(b-a\right)\overset{n}{\underset{k=0}{\sum }}c_{k}^{\left(n\right)}f\left(x\right)$$

$$C_{k}^{\left(n\right)}=\frac{{\left(-1\right)}^{n-k}}{k!\left(n-k\right)!n}\int_{0}^{n}\overset{n}{\underset{\begin{array}{c} j=0\\ j\neq k \end{array}}{\prod }}\left(t-j\right)dt$$

### 2.3 Polysomnography (PSG) and intermittent hypoxia index (IHI)

#### 2.3.1 PSG

We collected demographic and anthropometric characteristics of the study subjects, including gender, age, neck circumference, abdominal circumference, body mass index (BMI), and medical history. Sleep monitoring data were analyzed using Phillip G3 software. All patients included in the study underwent at least 8 h of overnight PSG. Standard electroencephalography (EEG) was used for PSG recordings, including frontal leads (F1, F2), central leads (C3, C4), occipital leads (O1, O2) and mastoid reference leads (M1, M2), electromyography (EMG), electrooculography (EOG) and monitoring of thoracoabdominal movements. Oxygen saturation was measured by pulse oximetry. Recordings and analyses were performed by experienced sleep lab technicians following the standard protocols recommended by the AASM. Apnea was defined as a drop of ≥ 90% of baseline in the peak signal of respiratory airflow monitored by nasal pressure, accompanied by persistent or enhanced inspiratory effort (thoracoabdominal movement present). Hypopnea was defined as a drop of ≥ 30% of baseline peak signal of respiratory airflow monitored by nasal pressure, lasting ≥ 2 breathing cycles, accompanied by ≥ 3% oxygen desaturation from pre-event baseline or with arousal. Regarding hypoxemia parameters, the lowest SpO_2_ (LSpO_2_) was the lowest oxygen saturation during sleep; T90 was the total sleep time spent with oxygen saturation below 90%; ODI was defined as the average number of desaturations per hour, if average oxygen saturation decreased ≥ 3% from baseline value, lasting for at least 10 s, it was counted as one desaturation event.

#### 2.3.2 IHI and calculation method

**Figure 2:**
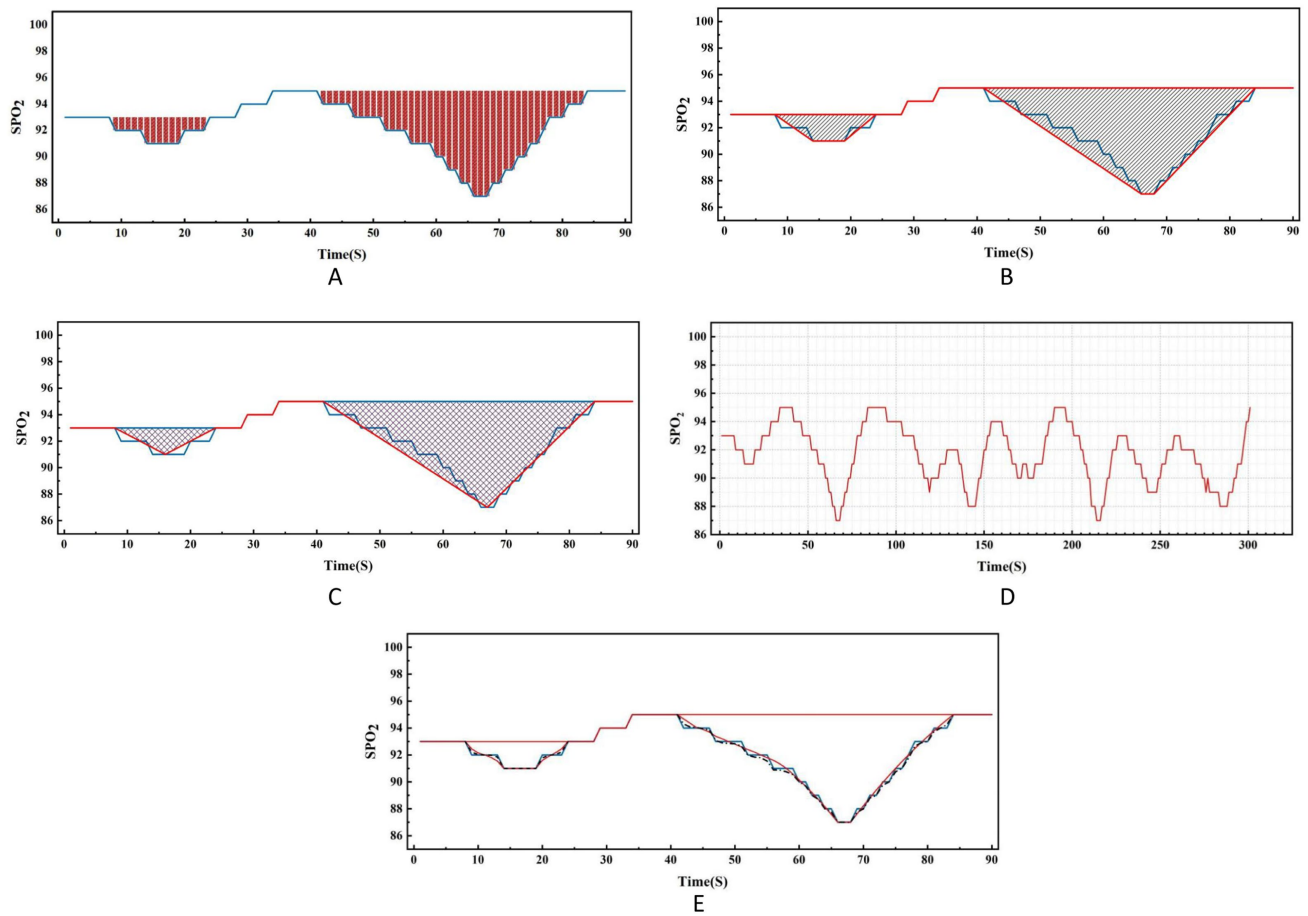
Comparison of nocturnal desaturation graph of OSA patient and various calculation methods.

**Figure 3:**
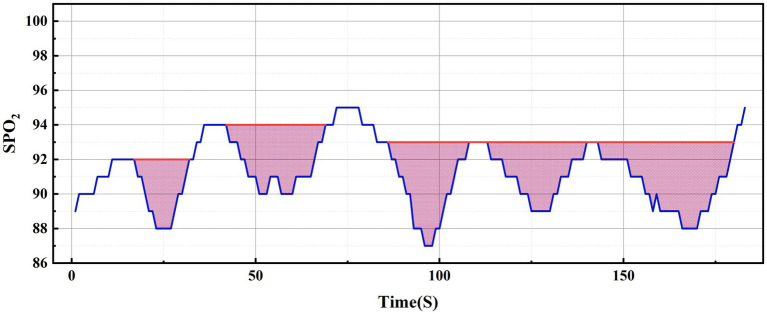
Graphical representation of the calculation of the intermittent hypoxia.

Regarding the concept of hypoxic load, the research literature reports so far have mainly used the rectangular formula (9), trapezoidal formula (2) or triangular formula (7, 10) methods to calculate the total area under the desaturation curve. The rectangular area formula involves individually selecting the values corresponding to each time point as the length and width of the rectangle, and finally summing the rectangular area of the time period (see Figure 2A); the trapezoidal area formula is similar to the rectangular area formula, but divides each figure into multiple small trapezoids, and finally sums the area (see Figure 2B). Wenhao (10) took the duration of apnea or hypopnea as the base, the depth of oxygen desaturation as the height, and approximated the desaturation graph as a triangle, using the triangular formula to calculate the total area under the desaturation curve (see Figure 2C). By observing the blood oxygen trend graph of PSG monitoring, it can be seen that the area under the desaturation curve formed when IH occurs at night in patients with OSA is mostly an irregular and dynamically-changing graph (see Figure 2D), and thus neither the triangular formula, trapezoidal formula, nor the rectangular formula can accurately reflect and calculate the actual hypoxic load in patients with OSA. In order to calculate the area under the desaturation curve more accurately, we tried different calculation methods, and finally, according to the characteristics of the nocturnal dynamic desaturation graphs of OSA patients, we chose the Newton-Cotes formula, which is a function formula with algebraic precision (see Figure 2E, the red line is the area under the desaturation curve calculated by the Newton-Cotes formula with lower algebraic precision, and the dashed line is the Newton-Cotes formula with higher algebraic precision; it can be seen that more accurate results can be obtained when the algebraic precision is high). The NLAS was developed to calculate the IHI by dividing the total area under the desaturation curve calculated using the Newton-Cotes formula by the TST in minutes (see Figure 3), and the NLAS was used to calculate the IHI by dividing the area under the desaturation curve by the TST in minutes. If the decrease in oxygen saturation (SpO_2_) was ≥ 3% of the baseline value, it was counted as one desaturation, with the time at which SpO_2_ began to fall as the starting point and the time at which oxygen saturation returned to its maximum value as the endpoint, and the graph of the starting point, endpoint, and the oxygen drop and reoxygenation delineating the area of desaturation was plotted (shaded in Figure 3). The portion of the curve where the SpO_2_ was less than 30% was regarded as the error of measurement and excluded.

### 2.4 Statistical analysis

Normally-distributed variables are expressed as mean ± standard deviation (X ± SD). Non-normally-distributed measurement data are expressed as quartile spacing M (P25, P75). Categorical data are described using frequency and percentage. A receiver operating characteristic (ROC) curve was used to calculate the threshold of IHI. The Pearson correlation coefficient was used to analyze the correlation between IHI and the hypoxic indicators ODI, T90, LSpO_2_ and the indicators representing the various dimensions of IH calculated by the software described above. *p* < 0.05 was considered statistically significant. All data analyses were performed using SPSS 26 statistical software (IBM SPSS Statistics for Windows, Armonk, NY, USA).

## 3 Results

### 3.1 General information

Table 2 presents the general information of the study subjects. A total of 732 patients with OSA were included in this study, with an average age of 44.58 years. Among them, there were 647 male patients (88.40%) and 85 female patients (11.60%). The mean values for neck circumference, waist circumference, and BMI were 40.61 cm, 102.53 cm, and 29.75 kg/m^2^, respectively. The median value of the AHI was 47.20, ranging from 5 to 138.3. The proportions of mild, moderate, and severe OSA were 14.10, 17.90, and 68%, respectively. The median values for ODI and time spent below 90% oxygen saturation (T90) were 41.30 and 23.60, respectively. The median value of the lowest oxygen saturation (LSpO_2_) was 76.00% (ranging from 30 to 94%). Based on the grouping of patients according to the lowest blood oxygen saturation, the proportions of mild, moderate, and severe hypoxia were 18.17, 17.90, and 58.73%, respectively.

**Table 2:** General information of study subjects.

Characteristics of study subjects (*n* = 732)	Numerical values
Age (years)	44 ± 13
Men, *n* (%)	647 (88.40%)
Women, *n* (%)	85 (11.60%)
neck circumference	40.61 ± 3.85
waist circumference	102.53 ± 17.69
BMI	29.75 ± 5.00
AHI (times per hour)	47.2 (24.1, 71.2)
Mild OSA, *n* (%)	103 (14.10%)
Moderate OSA, *n* (%)	131 (17.90%)
Severe OSA, *n* (%)	498 (68%)
ODI (times per hour)	41.30 (20.50, 69.70)
T90 (min)	23.60 (3.1, 111.35)
LSPO_2_%	76.00 (64.00, 84.00)
Non hypoxia, *n* (%).	38 (5.20%)
Mild hypoxia, *n* (%)	133 (18.17%)
Moderate hypoxia, *n* (%)	131 (17.90%)
Severe hypoxia, *n* (%)	430 (58.73%)

Note: Normally-distributed variables are expressed as the means ± SD, whereas the non-normally distributed measures are expressed as interquartile spacing M (P25, P75), and categorical data are described by frequencies and percentages. Mild OSA: 5 ≤ AHI ≤ 15; Moderate OSA: 15 < AHI ≤ 30; Severe OSA: AHI > 30; Non hypoxia: LSpO_2_ > 90%; Mild hypoxia: 85% ≤ LSpO_2_ < 90%; Moderate hypoxia: 80% ≤ LSpO_2_ ≤ 84%; Severe hypoxia: LSpO_2_ < 80%.

### 3.2 Calculation of IHI threshold value

**Figure 4:**
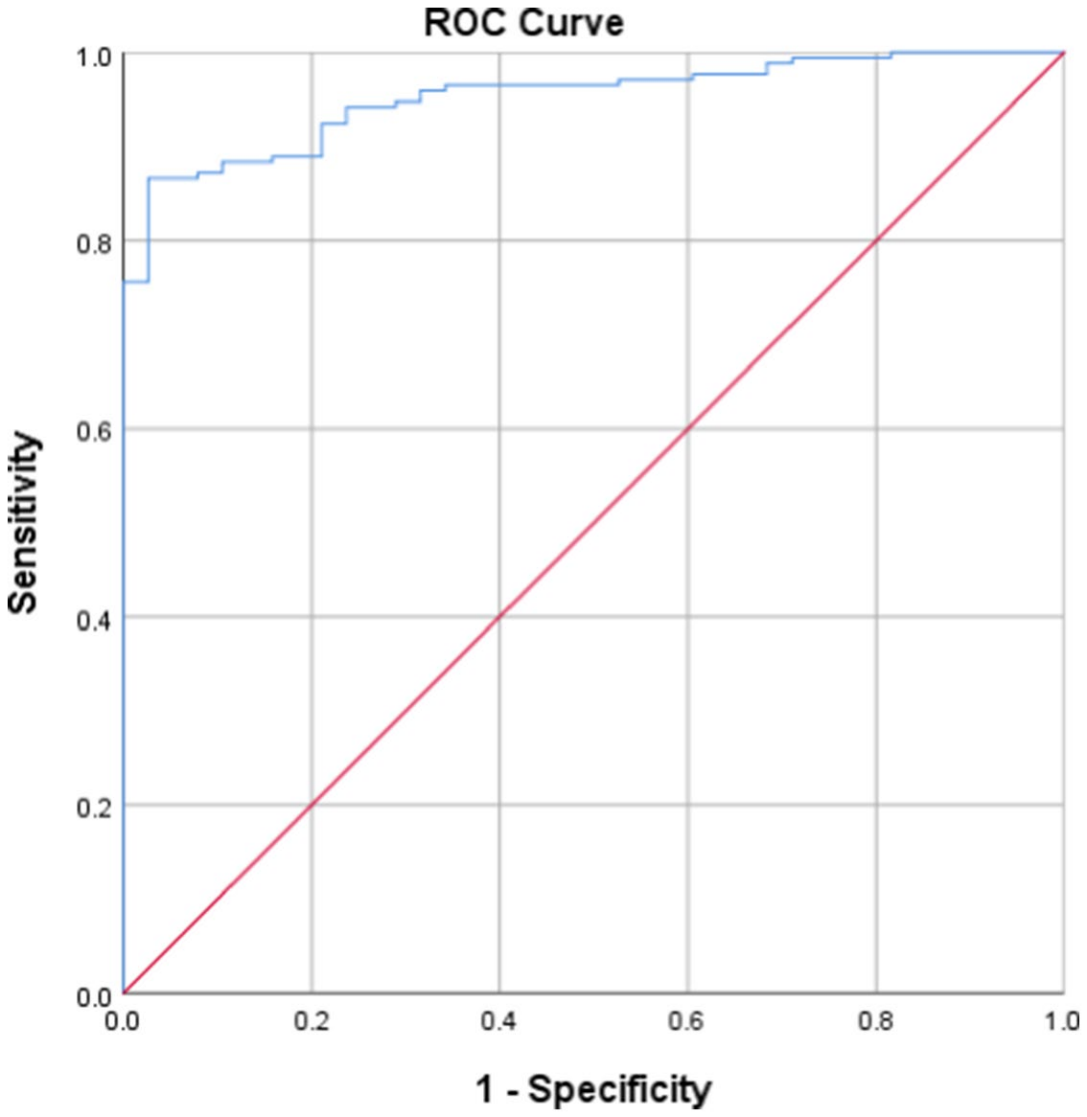
ROC Curve.

A lowest oxygen saturation value of less than 90% is considered a state of hypoxia (7, 11), that is, the IHI value corresponding to a minimum blood oxygen saturation of 90% is the threshold for determining whether a patient has hypoxia. Taking the current LSpO_2_ as the standard for judging the degree of hypoxia, the ROC curve of IHI and LSpO_2_ (Figure 4) had an AUC of 0.397 (95% confidence interval 0.913–0.960, *p <* 0.001, see Table 3), with an optimal cutoff value of 7.178. That is, the threshold for using IHI to diagnose intermittent hypoxia was 7.178 (%s/min).

**Table 3:** ROC curve analysis.

95% Confidence interval					
AUC	*p*-value	Lower limit	Upper limit	Odds ratio	Optimal cutoff value
0.937	<0.001	0.913	0.960	0.805	7.178

### 3.3 Correlation between IHI and PSG parameters

**Figure 5:**
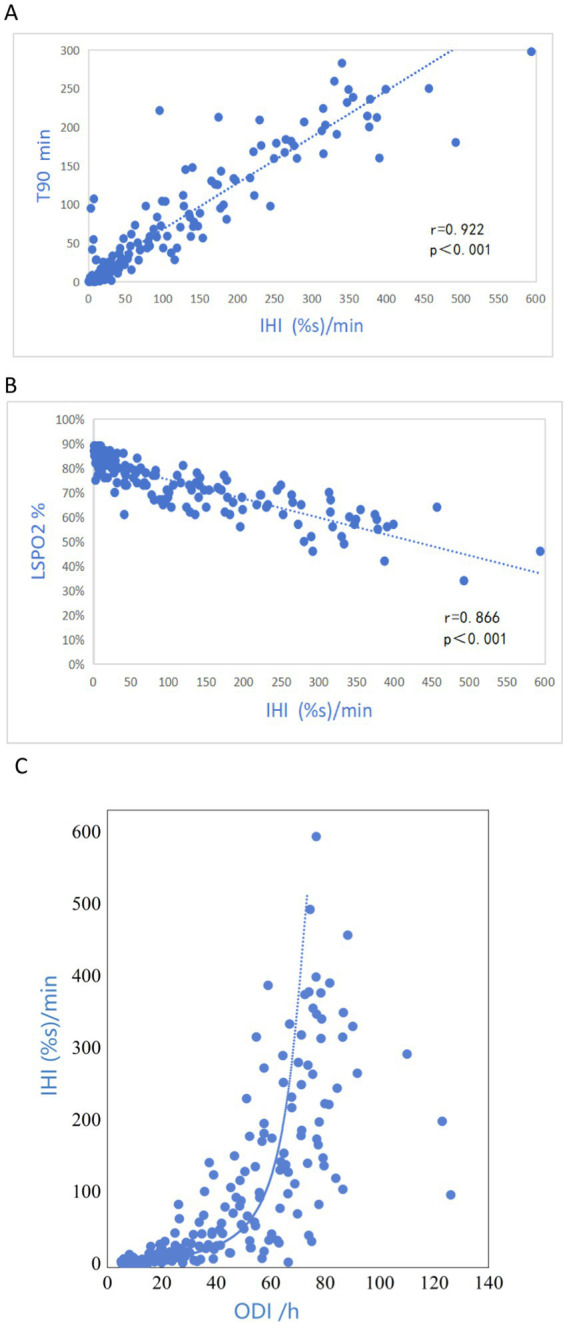
Scatterplot comparing the IHI and T90 **(A)**; LSpO_2_ **(B)**; ODI **(C)**.

Pearson’s chi-squared test showed that the participants’ IHI was strongly correlated with T90 (*r* = 0.922, *p* < 0.001, Figure 5A); there was a significant negative correlation between IHI and LSpO_2_ (*r* = 0.866, *p* < 0.001, Figure 5B); IHI also showed good correlations with ODI (*r* = 0.675, *p* < 0.001, Figure 5C).

## 4 Discussion

Currently, LSpO_2_, ODI, and T90 are the main clinical assessment indicators for determining the occurrence of IH and the severity of hypoxia in patients with OSA (6). Kainulainen et al. (11) found that the depth of desaturation negatively affects vigilance and the ability to maintain attention, which may lead to cognitive deficits in patients with OSA. ODI is the most widely used oxygen saturation parameter for assessing OSA and its co-morbidities (9), and is associated with subclinical atherosclerosis associated with OSA, all-cause mortality in heart failure, and post-procedural complications (6), as well as the QT variability index (QTVI), a measure of ventricular repolarization instability that predicts ventricular arrhythmias and sudden cardiac death (12). An article by Solhjoo (12) and others demonstrated that T90 is a predictor of QTVI. These studies suggest the need for an objective assessment of the degree of hypoxia in OSA in terms of the frequency, depth, and duration of desaturation in multiple dimensions.

To provide a more comprehensive assessment of nocturnal hypoxia in patients with OSA, Azarbarzin et al. (7) introduced the concept of hypoxic burden (HB). For clinical convenience, Fengwei Chen et al. (9) proposed the hypoxic burden index (HBI) as an alternative indicator to HB. Similar indices include hypoxic load (HL_100_) (2) and sleep breathing impairment index (SBII) (10). With these different measures, the degree of hypoxia in OSA patients cannot be accurately assessed clinically. However, all these indices have shortcomings in accurately calculating the total area under the desaturation curve. Researchers have mainly used rectangular, trapezoidal and triangular fixed graphical patterns to simulate and calculate the total area under the desaturation curve (2, 9, 10), but the area under the nightly desaturation curve in OSA patients is mostly irregular and dynamic, and the simulation method of fixed graphical patterns ignores and removes the correlation and fluctuation between the values. The rectangle method divides the area into numerous small rectangles, using individual values at each time point as the length and width of the rectangle. While it may seem precise, it does not consider the relationships between the numerical values. The trapezoid method faces similar issues, and the triangle method approximates the desaturation curve as a triangle, which can lead to significant errors. For example, Trzepizur et al. (13) reported that the median HB value they calculated was approximately 10 (% min)/h lower than that derived from a similar sample in the Sleep Heart Health Study (SHHS) conducted by the National Heart, Lung, and Blood Institute in the United States. There is a lack of standardized calculation criteria for HB, and given the graphical characteristics of dynamic changes in nocturnal IH shown under PSG monitoring, software is needed to assist in calculating the area under the desaturation curve (14). To address the shortcomings of previous calculation methods, our research team developed the Newton-Cotes formula-based NLAS. This formula allows for the calculation of the total area under the desaturation curve with different degrees of algebraic accuracy based on numerical changes. It reflects the integral situation of the correlation and fluctuation changes between data. Utilizing NLAS, we identified a new comprehensive evaluation index called the IHI, which simultaneously reflects information on the duration, frequency, and depth of desaturation, providing a more comprehensive characterization of the severity of IH in OSA patients.

Existing studies suggest that when oxygen saturation is above 90% and oxygen partial pressure exceeds 60 mmHg, mild decreases in blood oxygen should not impact cellular physiological functions and should not be considered as hypoxia (15, 16). The prognosis of OSA is closely associated with blood oxygen saturation falling below 90%, with SpO_2_ < 90% considered an independent predictor of mortality (17, 18). Therefore, in this study we defined hypoxia as having the lowest blood oxygen saturation below 90%. As mentioned above, IHI=S/TST, calculate the total area under the desaturation curve S by the following steps:

$$\int_{a}^{b}f\left(x\right)dx\approx \left(b-a\right)\overset{n}{\underset{k=0}{\sum }}c_{k}^{\left(n\right)}f\left(x\right)$$

$$C_{k}^{\left(n\right)}=\frac{{\left(-1\right)}^{n-k}}{k!\left(n-k\right)!n}\int_{0}^{n}\overset{n}{\underset{\begin{array}{c} j=0\\ j\neq k \end{array}}{\prod }}\left(t-j\right)dt$$

The parameters T90, LSPO2, and ODI are not included in the calculation of the IHI. They reflect individual pathophysiological characteristics of intermittent hypoxia, such as the duration, length, or frequency of oxygen desaturation. The IHI encompasses different dimensions of intermittent hypoxia indicators currently used in polysomnography for OSA patients and has a correlation with indicators such as T90, LSPO2, and ODI. The IHI is independent of existing intermittent hypoxia assessment indicators. This indicated that IHI, as a comprehensive evaluation index for IH, objectively reflected the severity of nocturnal hypoxia from multiple dimensions, including the duration, frequency, and depth of desaturation.

It is inescapable that this study has some limitations. First, our sample included more patients with severe OSA than other groups and the male-to-female ratio was unbalanced; second, the fact that the nocturnal minimum oxygen saturation in OSA patients was generally lower than 90% resulted in a higher positive rate of diagnosing hypoxia in patients using LSpO_2_, which may have caused a certain degree of error in the calculation of the threshold.

In summary, the IHI established based on NLAS covers different dimensions of hypoxia assessment indexes during sleep in OSA patients, and is expected to become an independent and comprehensive assessment index for nocturnal hypoxemia in OSA patients to compensate for the shortcomings of the existing IH assessment indexes.

## Data Availability

The raw data supporting the conclusions of this article will be made available by the authors, without undue reservation.

## References

[1] Martinez-Garcia MA Sánchez-de-la-Torre M White DP Azarbarzin A. Hypoxic burden in obstructive sleep apnea: present and future. Arch Bronconeumol. (2023) 59:36–43. doi: 10.1016/j.arbres.2022.08.005 36115739

[2] Thanaviratananich S Cheng H Chirakalwasan N Reutrakul S. Association between nocturnal hypoxemic burden and glucose metabolism. Sleep Breath. (2022) 26:1465–70. doi: 10.1007/s11325-021-02464-3, PMID: 34390444

[3] Rundo JV. Obstructive sleep apnea basics. Cleve Clin J Med. (2019) 86:2–9. doi: 10.3949/ccjm.86.s1.02, PMID: 31509498

[4] Demır N Öztura İ. New indices from polysomnographic measures for the severity of obstructive sleep apnea syndrome -a different look at obstructive sleep apnea syndrome. Noro Psikiyatr Ars. (2019) 57:222–7. doi: 10.29399/npa.23118, PMID: 32952425 PMC7481978

[5] Lam JC Mak JC Ip MS. Obesity, obstructive sleep apnoea and metabolic syndrome. Respirology. (2012) 17:223–36. doi: 10.1111/j.1440-1843.2011.02081.x, PMID: 21992649

[6] Cao W Luo J Xiao Y. A review of current tools used for evaluating the severity of obstructive sleep apnea. Nat Sci Sleep. (2020) 12:1023–31. doi: 10.2147/NSS.S275252, PMID: 33239929 PMC7680675

[7] Azarbarzin A Sands SA Stone KL Taranto-Montemurro L Messineo L Terrill PI. The hypoxic burden of sleep apnoea predicts cardiovascular disease-related mortality: the osteoporotic fractures in men study and the sleep heart health study. Eur Heart J. (2019) 40:1149–57. doi: 10.1093/eurheartj/ehy624, PMID: 30376054 PMC6451769

[8] Qingyang Li Nengchao Wang Dayi Yi. Numerical analysis. Beijing: Tsinghua University Press, (2008–2012). 100–103.

[9] Chen F Chen K Zhang C Chen X Huang J Jia P. Evaluating the clinical value of the hypoxia burden index in patients with obstructive sleep apnea. Postgrad Med. (2018) 130:436–41. doi: 10.1080/00325481.2018.1465326, PMID: 29676667

[10] Cao W Luo J Huang R Xiao Y. Implication of a novel measure of obstructive sleep apnea severity for cardiovascular morbidity. Sleep Med. (2023) 103:204–10. doi: 10.1016/j.sleep.2023.02.001, PMID: 36857991

[11] Kainulainen S Duce B Korkalainen H Oksenberg A Leino A Arnardottir ES. Severe desaturations increase psychomotor vigilance task-based median reaction time and number of lapses in obstructive sleep apnoea patients. Eur Respir J. (2020) 55:1901849. doi: 10.1183/13993003.01849-2019, PMID: 32029446 PMC7142879

[12] Solhjoo S Haigney MC Siddharthan T Koch A Punjabi NM. Sleep-disordered breathing destabilizes ventricular repolarization. Heart Rhythm. (2024) 22:808–16. doi: 10.1016/j.hrthm.2024.08.054 39214391

[13] Trzepizur W Blanchard M Ganem T Balusson F Feuilloy M Girault JM. Sleep apnea-specific hypoxic burden, symptom subtypes, and risk of cardiovascular events and all-cause mortality. Am J Respir Crit Care Med. (2022) 205:108–17. doi: 10.1164/rccm.202105-1274OC, PMID: 34648724

[14] Oldenburg O Costanzo MR Germany R McKane S Meyer TE Fox H. Improving nocturnal hypoxemic burden with Transvenous phrenic nerve stimulation for the treatment of central sleep apnea. J Cardiovasc Transl Res. (2021) 14:377–85. doi: 10.1007/s12265-020-10061-0, PMID: 32789619 PMC8043931

[15] Chaudhary B Dasti S Park Y Brown T Davis H Akhtar B. Hour-to-hour variability of oxygen saturation in sleep apnea. Chest. (1998) 113:719–22. doi: 10.1378/chest.113.3.719, PMID: 9515849

[16] Copstead LE Banasik JL. Pathophysiology (5th Edn.). Amsterdam (USA): Saunders, Elsevier. (2013). p. 486–487.

[17] Stone KL Blackwell TL Ancoli-Israel S Barrett-Connor E Bauer DC Cauley JA. Sleep disordered breathing and risk of stroke in older community-dwelling men. Sleep. (2016) 39:531–40. doi: 10.5665/sleep.5520, PMID: 26943468 PMC4763364

[18] Xie J Sert Kuniyoshi FH Covassin N Singh P Gami AS Wang S. Nocturnal hypoxemia due to obstructive sleep apnea is an independent predictor of poor prognosis after myocardial infarction. J Am Heart Assoc. (2016) 5:5. doi: 10.1161/jaha.115.003162, PMID: 27464791 PMC5015271

