# Supplementary material for: Intermittent hypoxia index: a new indicator for assessing the degree of intermittent hypoxia in obstructive sleep apnea
Source: Front Med (Lausanne). 2025 Apr 29;12:1400376. doi: 10.3389/fmed.2025.1400376 (PMC12069030; doi:10.3389/fmed.2025.1400376)
Supplement: Supplementary file 1 [file Data_Sheet_1.PDF]

NLAS user manual

NLAS is a computing software program that can be opened to all users after registration and login. After logging into the account, the patient's all-night oxygenation data can be uploaded, or historical monitoring data can be selected, and the total sleep time captured in the sleep monitoring report can then be entered into the appropriate location. Clicking on the Start button produces data on the total area, average area, average duration, average desaturation depth and intermittent hypoxia index (IHI) under the desaturation curve. It is also possible to see the oxygenation distribution throughout the night, the total area under the desaturation curve, and the IHI.

IHI calculation method

① Determination of desaturation event: if there was an oxygenation decrease of $\geq 3\%$, this was defined as a desaturation event.

② Determination of the start point and the end point of desaturation events: in a desaturation event, the time at which SpO_2 began to fall was regarded as the start point and the time at which oxygen saturation returned to its maximum value as the end point. For events where the oxygenation did not return to baseline, the start and end points were determined using the mean oxygen saturation as the baseline.

③ Determination of the desaturation area: the graph area between the oxygen drop curve, reoxygenation curve and baseline saturation formed the area under the desaturation curve, and the sum of all the desaturation areas constituted the total area under the overnight desaturation curve.

④ Calculation of the total area under the desaturation curve: the area under the desaturation curve formed by IH at night in OSA patients was mostly an irregular dynamic pattern.

Therefore, we used a new calculation method, using the Newton-Cotes formula to calculate the area under the desaturation curve, applying the following steps to calculate the total area under the desaturation curve S:

$$\int_a^b f(x)dx \approx (b-a)\sum_{k=0}^{n} c_k^{(n)} f(x)$$

$$C_k^{(n)} = \frac{(-1)^{n-k}}{k!\,(n-k)!\,n}\int_0^n \prod_{\substack{j=0\\ j\neq k}}^{n} (t-j)dt$$

where [A, B] is the integral interval, "n" is the constant fraction, F(x) is the integrand, "k" is the quadrature node, $C_k^{(n)}$ is the Cotes coefficient, "T" is the independent variable of the integrand, dt and dx denote the increments of independent variables of integrand function, J denotes the integral independent variable in the 0-n interval.

As can be seen from the formula, the Newton-Cotes formula has multiple algebraic precision (see figure below for different algebraic precision figures). Therefore, it has good stability and a small error.

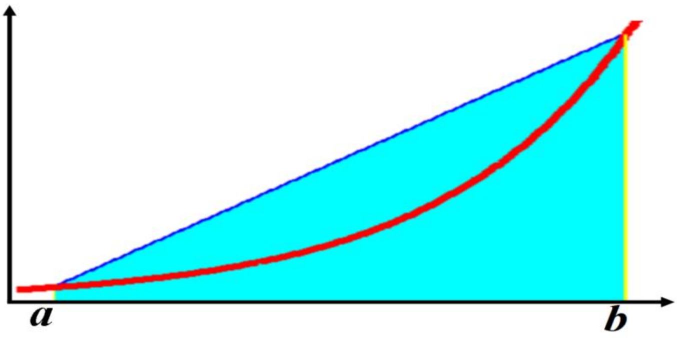

When n = 1, it is a trapezoid formula: one-order algebraic precision

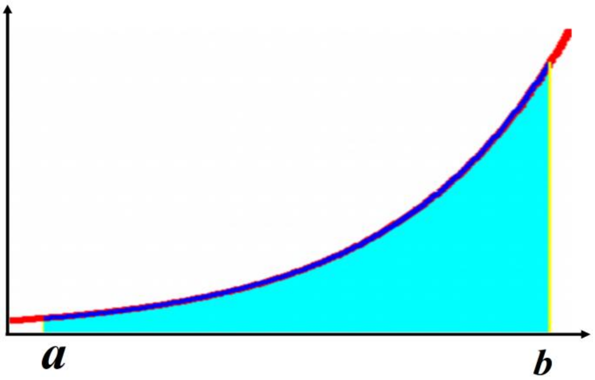

N = 4,Cotes formula: fifth-degree algebraic precision

(A) the trapezoidal formula and (B) the Newton-Cotes formula

⑤ Calculation of IHI: IHI = total area under the desaturation curve in minutes (total sleep time: TST) calculated using the Newton-Cotes formula. The TST is obtained from a report on sleep monitoring.

⑥ Output IHI: after logging on to NLAS, the patient's overnight oxygenation data and TST were uploaded, then the output IHI was calculated by clicking the Start button.

IHI = S/TST, where S represents the total area under the desaturation curve and TST represents the total sleep time in minutes,
